# Comprehensive Transcriptome and Metabolic Profiling of Petal Color Development in *Lycoris sprengeri*

**DOI:** 10.3389/fpls.2021.747131

**Published:** 2021-12-03

**Authors:** Feng Yang, Chao-han Li, Debatosh Das, Yu-hong Zheng, Tao Song, Lan-xiang Wang, Mo-Xian Chen, Qing-zhu Li, Jianhua Zhang

**Affiliations:** ^1^Forestry and Pomology Research Institute, Protected Horticultural Research Institute, Shanghai Academy of Agricultural Sciences, Shanghai, China; ^2^Shenzhen Research Institute, The Chinese University of Hong Kong, Shenzhen, China; ^3^Ornamental Plant Research Center, Institute of Botany, Jiangsu Province and Chinese Academy of Sciences (Nanjing Botanical Garden Memorial Sun Yat-Sen), Nanjing, China; ^4^Shenzhen Institute of Synthetic Biology, Shenzhen Institutes of Advanced Technology, Chinese Academy of Sciences, Shenzhen, China; ^5^Department of Biology, Hong Kong Baptist University, and State Key Laboratory of Agrobiotechnology, The Chinese University of Hong Kong, Hong Kong SAR, China

**Keywords:** *Lycoris sprengeri*, flavonoids, transcriptome, anthocyanin, petal color, metabolome

## Abstract

*Lycoris sprengeri* (*L. sprengeri*) is an important ornamental bulbous plant, and its numerous varieties in different color forms are widely planted. Multiple color types of petals in *L. sprengeri* provide us with possibilities to delineate the complicated metabolic networks underlying the biochemical traits behind color formation in this plant species, especially petal color. In this study, we sequenced and annotated a reference transcriptome of pink and white petals of *L. sprengeri* and analyzed the metabolic role of anthocyanin biosynthesis in regulating color pigment metabolism. Briefly, white and pink petal samples were sequenced with an Illumina platform, to obtain the reads that could be assembled into 100,778 unique sequences. Sequences expressed differentially between white vs. pink petals were further annotated with the terms of Gene Ontology (GO), Clusters of Orthologous Groups (COG), Kyoto Encyclopedia of Genes and Genomes (KEGG), and eggNOG. Gene expression analyses revealed the repression of anthocyanin and steroid biosynthesis enzymes and R2R3 MYB transcription factor (TF) genes in white petals compared to pink petals. Furthermore, the targeted metabolic profiling of anthocyanins revealed that color-related delphinidin (Del) and cyanidin (Cy) pigments are lower in white petals, which correlate well with the reduced gene expression levels of anthocyanin biosynthesis genes. Taken together, it is hypothesized that anthocyanin biosynthesis, steroid biosynthesis, and R2R3 MYB TFs may play vital regulatory roles in petal color development in *L. sprengeri*. This work provides a valuable genomic resource for flower breeding and metabolic engineering in horticulture and markers for studying the flower trait evolution of *L. sprengeri*.

## Introduction

Genus *Lycoris* belongs to the Amaryllidaceae family, which consists of approximately 20 species of flowering plants and is native to the moist warm temperate woodlands of eastern and southern Asia, nearly 15 of which (10 endemics) are spread throughout China (Ren et al., 2021). Most of the *Lycoris* species are commonly cultivated as bulbous plants in countries such as China, Korea, Japan, and Vietnam (Ping-Sheng et al., 1994; Shi et al., 2006). In comparison to other well-known bulbous flowers, such as *Narcissus* spp. and *Lilium* spp., *Lycoris* has its specific traits and benefits. For example, it flowers at a time of the year during which others are not active. These flowers are characterized by their pastel, plentiful colors, and multiple flower shapes (Ping-Sheng et al., 1994). Thus, globally *Lycoris* species, such as *L. radiata*, *L. aurea*, and *L. sprengeri*, have been very popular as ornamental plants in the past few decades (Zhou et al., 2007). With an increasing demand for *Lycoris* as a commercial horticultural product, the breeding of different varieties with new petal forms and/or colors has become a valued necessity for *Lycoris.* Moreover, the bulbs of *Lycoris* have been used in traditional Chinese medicine (TCM) as some Amaryllidaceae alkaloids isolated from the bulbs of *Lycoris* have been reported to exhibit immunostimulatory, antitumor, antiviral, and antimalarial activities (Jin, 2009; Son et al., 2010; Wang et al., 2013).

**FIGURE 1:**
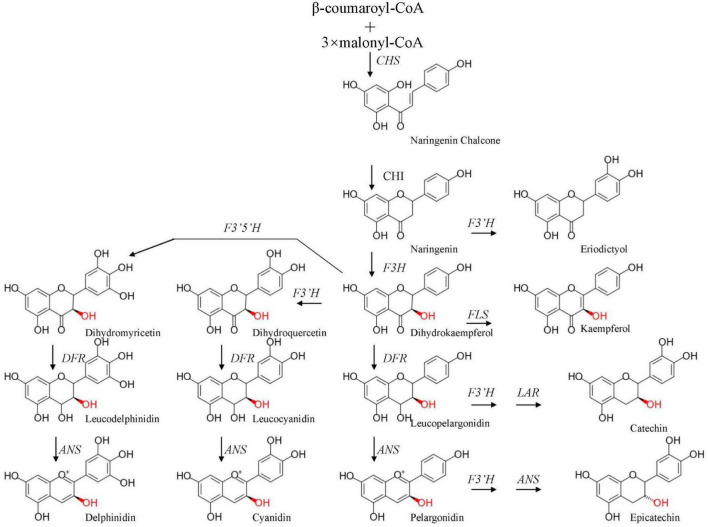
Simplified model of the flavonoid pathway, comprising the general phenylpropanoid pathway, the anthocyanin branch, and other subgroups of flavonoid end products. CHS, chalcone synthase; F3H, flavanone 3-hydroxylase; DFR, dihydroflavonol-4-reductase; ANS, anthocyanidin synthase; F3′H, flavonoid 3′-hydroxylase; FLS, flavonol synthase; F3′5′H, flavonoid 3′,5′-hydroxylase; LAR, leucoanthocyanidin 4-reductase.

The flower petal color of *L. sprengeri* varies widely from pure white color (with only the faintest pale purple stripe on the abaxial base of the petals) to an intense pink color (with blue color on the tips) (Cai et al., 2020). Flower pigmentation is mainly caused by the accumulation of pigments such as flavonoids, carotenoids, and betacyanin within epidermal cells (Zhao et al., 2012). Flavonoids consist of chalcones, anthoxanthins, and anthocyanin. Anthocyanin biosynthesis *via* flavonoid metabolism has been studied on flower development because of its high antioxidant content and UV protection properties in plants (Ithal and Reddy, 2004; Irani and Grotewold, 2005; Paśko et al., 2009; Ordidge et al., 2012; Feng et al., 2013). Anthocyanin pigment is the main pigment in flowering plants, and anthocyanin accumulation is tightly linked to floral development, including petal color changes (Weiss, 2000). Anthocyanin biosynthesis is catalyzed by two groups of genes, namely, structural genes and functional regulatory genes. The first group of structural genes includes flavanone 3-hydroxylase (*F3H*), chalcone synthase (*CHS*), anthocyanidin synthase (*ANS*), dihydroflavonol-4-reductase (*DFR*), flavonoid 3′-hydroxylase (F3′H), flavonoid 3′,5′-hydroxylase (F3′5′H), etc., which represent the enzymes responsible for the biochemical reactions of anthocyanin synthesis (Tanaka et al., 2008; Huang et al., 2012; Tan et al., 2013; Figure 1). DFR could catalyze a stereospecific reduction of the three dihydroflavonols to leucoanthocyanidins while flavonol synthase (FLS) catalyzes the conversion of dihydrokaempferol and dihydroquercetin to a variety of copigment flavanols and glycosidic derivatives that determine the petal color (white and/or pink) (Luo et al., 2015). The second group includes regulatory genes such as transcription factors (TFs), which regulate the expression of the abovementioned structural genes. It has been reported that the most important TFs for anthocyanin biosynthesis belong to the basic helix-loop-helix (*bHLH*), *MYB*, and *WD40* gene families (Zhou et al., 2012; Li et al., 2013). The coordinated expression of structural genes and TFs may lead to differential anthocyanin biosynthesis and accumulation during petal color development.

In recent years, transcriptome mapping has become a particularly effective method for genome annotation and discovery and the annotation of novel functional genes, especially in non-model plants, which lack a reference genome (Wang et al., 2009; Song et al., 2020). Sequencing technologies have dramatically accelerated genome-wide transcriptome studies and have been widely used to explore a novel gene structure and gene expression, even in plants without a reference genome (Chen et al., 2011; Liu et al., 2011; Shen et al., 2011). In previous studies related to *Lycoris*, the transcriptome sequencing analysis was performed to investigate the molecular basis of the synthesis of Amaryllidaceae alkaloids in *L. aurea* and *L. longituba* species (Wang et al., 2013; Park et al., 2019; Li et al., 2020). However, very few systematic studies have been performed to explore the molecular mechanism in the regulation of pigment accumulation in the different color forms of *Lycoris* petals. In this study, a comparative transcriptome and metabolome analysis of the gene expression and metabolite accumulation between white and pink petals of *L. sprengeri* was first carried out using Illumina sequencing and targeted metabolic profiling with the liquid chromatographic tandem mass spectrometry (LC-MS/MS) method, respectively. Thereby, we mainly focus on the identification of structural genes in anthocyanin biosynthesis, for example, enzymes, regulatory gene TFs, and on the differentially accumulating metabolites that may coregulate the observed color variation in *L. sprengeri* petals. The assembled and newly annotated transcriptome resource for *Lycoris* petals will provide an additional overview of genes involved in pigment pathways, including flavonoid biosynthesis. In summary, this work would enable us to understand the molecular changes behind the flower color variation in *L. sprengeri*.

## Materials and Methods

### RNA Isolation, Library Preparation, and Illumina Sequencing

*Lycoris sprengeri* plants for this study were collected by Prof. Zheng Yu-hong (Institute of Botany, Chinese Academy of Sciences, Jiangsu Province, China). Voucher specimens of *L. sprengeri* (0653967) were deposited at the herbarium of the Institute of Botany, Chinese Academy of Sciences. A total amount of 1 μg isolated RNA per sample was used as input material for sequencing. RNA sequencing (RNA-seq) libraries were constructed using the NEB Next^®^Ultra^TM^ RNA Library Prep kit for Illumina^®^ (NEB, Ipswich, MA, United States) following the manufacturer’s recommendations as described. Briefly, poly-T oligo magnetic extraction of messenger RNA (mRNA) from the isolated total RNA was carried out, followed by its fragmentation using divalent cations and heating in NEB Next Frist Strand synthesis reaction buffer and first strand synthesis with random hexamer and M-MuLV Reverse Transcriptase. Following this, a second strand cDNA synthesis was performed on the first strand with DNA Polymerase I and RNase H. In the process, the leftover overhangs in the second cDNA were filled *via* exonuclease/polymerase activities. After adenylation of 3’ ends of DNA, NEBNext Adaptor carrying hairpin loop structure was ligated for hybridization. A 240-bp-long cDNA fragments were selected preferably by purifying the library fragments with the AMPure XP system (Beckman Coulter, Beverly, MA, United States). Following this, 3 μl USER Enzyme (NEB, Ipswich, MA, United States) was incubated with the abovementioned adaptor-ligated and size-selected cDNA, first for 15 min at 37°C followed by 5 min at 95°C. Following this, PCR was carried out using Phusion High-Fidelity DNA polymerase and Universal PCR primers and Index (X) Primer. At last, again AMPure XP system (Beckman Coulter, Beverly, MA, United States) was used to purify the extract of the synthesized PCR products. In the final step, the quality check of the constructed library was assessed using an Agilent Bioanalyzer 2100 system (Agilent, Santa Clara, CA, United States) (Lou et al., 2014). Samples were subsequently sequenced on an Illumina HiSeq platform.

### Transcriptome Assembly, Annotation, and Differentially Expressed Genes (DEGs) Analyses

The raw reads obtained earlier were filtered to remove adaptors and reads where unknown nucleotides exceeded 5%. The filtered clean reads were *de novo* assembled with Trinity^1^ using the following parameters: “K-mer = 25 and group pairs distance = 300” (Grabherr et al., 2011). Briefly, the assembly process was as follows: short reads from sequencing were assembled into longer contigs based on sequence overlaps. Following this, the different contigs from another transcript and the distance between them were further recognized by mapping the clean reads back to the corresponding longer contigs based on their paired-end information. In this way, the sequence of different transcripts was obtained. Finally, these potential transcript sequences were clustered (TGI Clustering) to obtain unitranscripts (Pertea et al., 2003). To identify a uni-transcript identity, they were aligned to multiple protein databases in BLASTx (*E*-value ≤ 10^––5^), including NCBI non-redundant (Nr^2^), Swiss-Prot^3^, Trembl, Kyoto Encyclopedia of Genes and Genomes (KEGG^4^), Clusters of Orthologous Groups (COG^5^), euKaryotic Orthologous Groups (KOG^6^), eggNOG (v4.5^7^), Pfam (Protein family^8^), and Gene Ontology (GO^9^) databases. SOAP aligner^10^ was used to realign all usable reads to each uni-transcript to evaluate the depth of data coverage.

This normalization was post-implemented to obtain reads per kilobytes per million reads (FPKM; Mortazavi et al., 2008). Afterward, uni-transcript abundance differences between white and pink petals were calculated based on the ratio of the FPKM values (Benjamini and Yekutieli, 2001). Here, prior to the differential gene expression analysis, read counts were adjusted, and the differential expression analysis of white vs. pink petals was performed using the EBSeq R package (Leng et al., 2015). The cutoff of | log_2_(fold change)| ≥ 1 and false discovery rate (FDR) ≤ 0.01 was set as the threshold cutoff for identifying DEGs.

### Kyoto Encyclopedia of Genes and Genomes and Gene Ontology Enrichment Analyses

Kyoto Encyclopedia of Genes and Genomes (Kanehisa et al., 2007) was used for the characterization of enriched metabolic pathways (based on gene expression) in white vs. pink petals. KOBAS statistical test pipeline (Xie et al., 2011) was used to identify significantly overrepresented metabolic pathways for DEGs between white and pink petals. Unique sequences were represented with EC numbers for which BLASTX (against KEGG) scores were associated with *E*-value ≤ 10^–5^ and based on this, these unique sequences were mapped to match the specific biochemical pathways (according to the corresponding KEGG EC distribution).

To gain insights into the functional enrichments in DEGs or gene clusters, we conducted the GO enrichment test to find the overrepresented GO terms in gene lists using BiNGO (Maere et al., 2005). The *p*-values were adjusted using Benjamini and Hochberg’s (1995) FDR correction method for multiple testing. GO terms with FDR ≤ 0.01 and consisting of at least five genes were considered as significantly enriched.

### Quantitative PCR Analysis of Differentially Expressed Genes and Transcription Factors Identified in RNA-Seq Approach

All the petal color implicated unitranscripts differentially expressed in white vs. pink petals were subjected to real-time quantitative PCR (qPCR) with gene-specific primers (Supplementary Table 10). cDNA synthesis and qPCR were performed as described previously (Qi et al., 2013) on a MyiQ Single-Color Real-Time Detection System (Bio-Rad, Watford, United Kingdom). *LsUbiquitin* was used as a housekeeping gene.

### Extraction and Quantification of Metabolites

Anthocyanin metabolites were determined according to the following method: 0.5 g white and pink with blue points of petal tips (pink), which are at the full-bloom stage were weighed and ground into powder in liquid nitrogen. About 7.5 ml of the extract was added and ultrasonically treated for 0.5 h in a 4°C water bath and centrifuged at 10,000 *g* under 4°C for 5 min. Subsequently, the supernatant was transferred to a new tube. A 5 ml of concentrated HCl was added and incubated at 90°C for 40 min, and the volume was finally fixed to 20 ml. After filtration with a 0.22-μm membrane, LC-MS/MS detection was performed. Metabolite contents were determined using Agilent 1290 high performance liquid chromatography (Aglient1290) in tandem with AB QTRAP6500 mass spectrometer (SciEx-6500QTRAP), and poroshell column 120 SB-C18 RP (2.1 × 150, 2.7 μm) was used in this approach.

To measure the contents of about 105 kinds of flavonoid-related metabolites and phenolic compounds, a broad-target metabolic analysis was employed to analyze an overview of metabolic changes between white and pink petals using LC-MS/MS. Briefly, 0.2 g of the petal was freeze-dried, and a 600 μl mixture of methanol and water (2/1, V/V) and 400 μl chloroform were added to each sample. The mixtures were ultrasonicated in an ice-water bath for 20 min, then the samples were centrifuged at 13,000 rpm for 10 min at 4°C. A 500-μl supernatants were removed to a new tube, and a 400 μl mixture of methanol and water (2/1, V/V) was added to the residue. The mixtures were ultrasonicated at an ice-water bath for 20 min, then the samples were centrifuged at 13,000 rpm for 10 min at 4°C. A 300-μl supernatants were combined into a new tube (total 800 μl). A 200-μl supernatants were vacuum-dried and redissolved in 200 μl of water-methanol (V/V = 18:7) for the LC-MS/MS analysis. A 2-chloro-L-phenylalanine (12 ng/ml) was used as an internal standard. All of the standard chemicals were purchased from Sigma-Aldrich. High performance liquid chromatography (HPLC) grade solvents were ordered from Thermo Fisher Scientific (Waltham, MA, United States). Metabolite analysis was performed with an AB ExionLC (AB Sciex, Framingham, MA, United States) equipped with a Waters UPLC HSS T3 column (100 mm × 2.1 mm, 1.7 μm). LC elution was monitored using a Qtrap 6500+ mass spectrometer (AB Sciex, Framingham, MA, United States) operating in a positive or negative detection mode. The binary gradient elution system consisted of (A) acetonitrile and (B) water (containing 0.1% formic acid, v/v). Separation was achieved using a gradient: 0 min, 0 B; 2 min, 0 B; 36 min, 55% B; 39 min, 95% B; 42 min, 95% B; 42.1 min, 0 B; and 45 min, 0 B. The flow rate was 0.30 ml/min. The main metabolites that may affect flavonoid compositions were quantified by using the following mother and daughter ion pair: delphinidin (Del) 303→229, epicatechin (Ep) 289→109, cyanidin (Cy) 287→231, catechin (Ca) 290→109, myricetin (My) 317→151, eriodictyol (Er) 287→151, and kaempferol (Km) 285→229. Experiments were conducted with three independent biological replicates.

### Measurements of Brassinolide in White and Pink Petals in *Lycoris sprengeri*

All the petal samples were ground with precooled mortar in liquid nitrogen, and about 1.0 g of powder for each sample was transferred into fresh tubes. Precooled 95% methanol was added to the samples, and the samples were incubated at 4°C for 2 h. After that, the centrifugation was performed at 10,000 *g* under 4°C for 5 min, the supernatant was taken and precipitation was performed repeatedly, and supernatants were combined. The sample was directly loaded onto the preloaded column (18 ml liquid was taken) and eluted with 3 ml methanol. Methanol was blow-dried with nitrogen, dissolved with 300 μl methanol, filtered through a 0.22-μm membrane, and examined by HPLC and tandem mass spectrometry (HPLC-MS/MS).

For the quantification of brassinolide (BL), 250 pg BL (Sigma-Aldrich, St. Louis, MO, United States) was used together as the standard with the extracts. All samples were injected onto a poroshell 120 (2.1 × 150, 2.7 μm) reverse phase column. The inlet method was set as follows: mobile phase A, methanol and B, 0.1% ammonium hydroxide in ddH_2_O. Gradient: 0–2 min, 80% A; 2–3.5 min, 80–85% A; 3.5–6 min, 95% A; 6–6.1 min, 95–80% A; and 6.1–10 min, 80% A. BL was detected in an ESI source performed in a positive mode of MRM detection. The ESI parameters were set as follows: air curtain gas: 15 psi; spray voltage: +4,500 V, −4,000; atomizing gas pressure: 65 psi; auxiliary gas pressure: 70 psi; atomization temperature: 350°C.

### Measurements of pH Values in White and Pink Petals in *Lycoris sprengeri*

The measurement of pH value between white and pink petals was performed as follows: about 1 g of fresh petals were mixed with 0.1 g of quartz sand and then fully ground in liquid nitrogen. Centrifugation was performed at 1,200 r⋅min^–1^ at 4°C for 1 min, and the supernatant was quickly transferred to a new 1.5-ml centrifuge tube. Immediately, a flat pH meter (pH5F, Shanghai Sanxin Instrument Company, Shanghai, China) was used to measure the pH value of the supernatant at room temperature. The three different sample replicates that were used from white and pink petals were taken, and each sample was repeated at least four times.

## Results

### Phenotypic Characteristic Analysis of Pink and White Petals in *Lycoris sprengeri*

**FIGURE 2:**
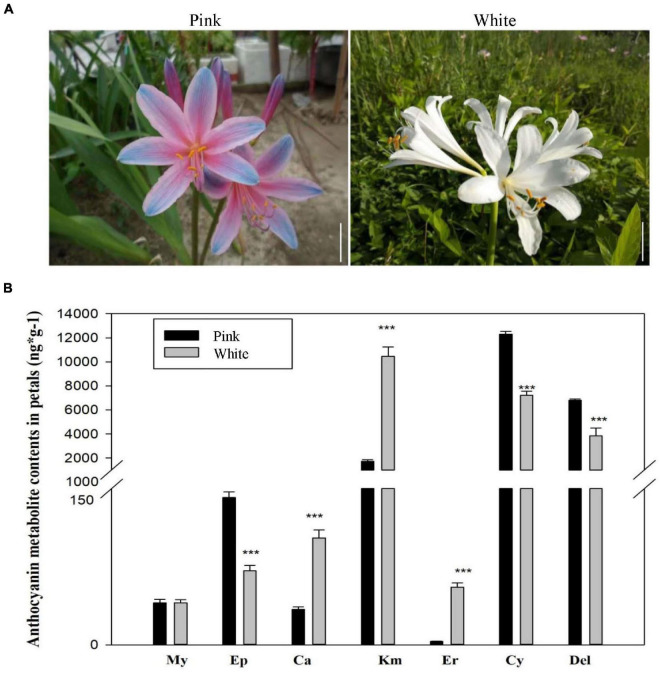
Phenotypic characteristics analysis of pink and white petals in *Lycoris sprengeri*. **(A)** Morphological comparison of white and pink petals of *L. sprengeri* under wild conditions. **(B)** Liquid chromatographic tandem mass spectrometry (LC-MS/MS) analysis of core metabolites in flavonoid biosynthesis process.

Though various studies have been performed to study the flower bud differentiation in *Lycoris* plants, limited investigations on the mechanisms of the color difference of Lycoris flowers were employed. In this study, the two contrasting colors of *L. sprengeri* petals, namely, white and pink petals, at full-bloom stages were obtained (Figure 2A) and the color difference prompts us to hypothesize that anthocyanin biosynthesis or other pigments biosynthesis was different from each other in *L. sprengeri*. The targeted LC-MS/MS results of the pigments-related metabolites suggested that major flavonoid biosynthesis metabolites, which may be involved in flower color development, have different accumulation levels (Figure 2B). For example, the accumulation levels of Del, Ep, and Cy are significantly lower in white petals than that in pink petals whereas the accumulation levels of Ca, Er, and Km were significantly higher in white petals than those in pink petals. As expected, pink *L. sprengeri* petals contained high levels of two anthocyanin compounds responsible for color pigmentation: Del and Cy. In contrast, significantly reduced levels of color anthocyanins and a few derivatives were detected in the white petals of *L. sprengeri* (Figure 2B). In addition, the accumulation levels of My in both pink and white petals were similar. To obtain the general overview of pigment metabolite levels, the composition of common copigment flavonoids, such as quercitrin, cyanin chloride, Del 3-glucoside, caffeic acid, 4-hydroxycinnamic acid, and other 105 metabolites in total for pink and white petals has been provided (Supplementary Table 6).

### Sequencing and Transcriptome Assembly

**FIGURE 3:**
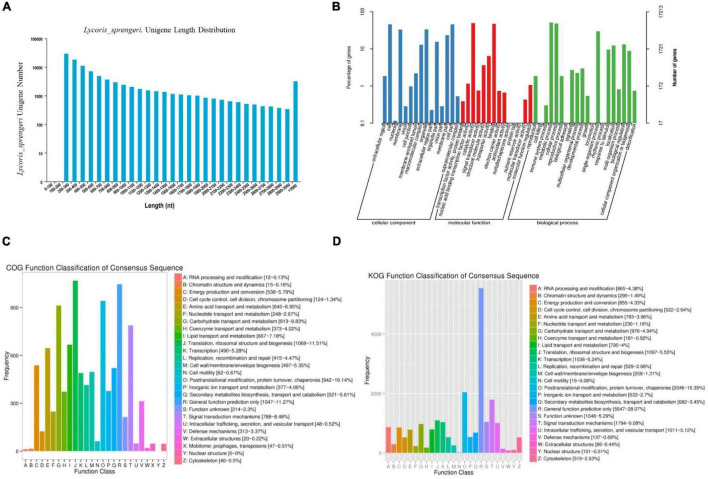
An overview of the RNA sequencing (RNA-seq) data analysis in white and pink petals. **(A)** Unigene length distribution of sequencing reads and contigs in *L. sprengeri* RNA-seq. **(B)** Histogram representing Gene 02Ontology (GO) classification. GO categories, shown on the *x*-axis, were grouped into three main ontologies: biological process, cellular component, and molecular function. The right *y*-axis indicates the number of genes in each category, while the left *y*-axis indicates the percentage of total genes in that category. The “all gene” indicates that unigenes were those assembled from reads from pink and white petals. **(C)** Histogram representing clusters of orthologous groups (COG) classification. A total of 33,456 unigenes were assigned to 24 categories in the COG classification. **(D)** Histogram representing the eggNOG classification. The *y*-axis “frequency” indicates the number of genes in a specific functional cluster. The right side of the legend shows a description of the 24 functional categories.

To investigate the molecular basis regarding color development in *L. sprengeri* petals, genome-wide transcriptome analysis is employed to analyze the transcriptomic gene expressions. At first, the two cDNA libraries from white and pink petals were prepared in Illumina sequencing from three biological replicates per sample, which generated 25,822,803 sequences with 7,743,689,931 bp from white petals and 24,664,320 sequences with 7,378,331,053 nucleotides (basepair) from pink petals. After the removal of low-quality raw reads, which included too short, empty, too many Ns, we obtained an average of 25,822,803 (white) and 24,664,320 (pink) high-quality sequences, respectively (Supplementary Table 1). Transcriptome data obtained hereby have been uploaded to the Sequence Read Archive^11^ and can be accessed with the accession number PRJNA714286. These reads were assembled into 34,336 total genes, 100,778 total isogenes, and 203,657,668 total reads with an average length of 1,037.74 bp. In total, 34,336 unique sequences were annotated using different databases (Supplementary Table 2). Unique sequences that were not among the GenBank sequences were considered to be the novel transcripts of *L. sprengeri*. The number of isogenes with over 1.0 kb length was 21,621, and the number of isogenes with less than 300 bp length was 28,908 (Supplementary Table 3). An overview of the sequence length distribution is shown, which displays good data assembly performance due to an optimal N50 (Figure 3A and Supplementary Table 3). A principal component analysis (PCA) suggested close clustering of replicates within the samples and hence obtaining excellent sample data reproducibility (Supplementary Figure 1). We suggest that a large quantity of unique sequences from *L. sprengeri* petals should cover a vast majority of genes in this species. This should provide, for the first time, a powerful gene expression resource for this medicinal and ornamental plant.

### Functional Annotation

To analyze and annotate the identified unigenes, unigenes were searched against different databases. A 34,336 spliced *L. sprengeri* transcripts were assigned with GO terms based on sequence similarity with already known proteins and corresponding GO slim terms from TAIR. Among the 34,336 spliced transcripts, 17,213 annotated sequences could be annotated with GO terms while 17,123 lacked it (Figure 3B). GO annotations of unique sequences could be categorized into molecular function (37,971 unique sequences), biological process (19,342 unique sequences), and cellular component (33,505 unique sequences) (Figure 3B). GO term annotation provided a broad overview of the functional groups of genes cataloged in our *L. sprengeri* petal transcriptome.

To further examine the integrity of our transcriptome library and the effectiveness of the annotation process, we identified the unigene numbers based on the COG and eggNOG classification. Altogether, there were 9,395 unigenes identified by the COG (Figure 3C) and 19,760 unigenes by the eggNOG classification (Figure 3D). Among the 24 COG categories, the cluster of “Translation, ribosomal structure, and biogenesis” accounted for the largest proportion of unigenes (1,069, 11.51%) followed by “General function prediction only” (1,047, 11.27%), “Post-translational modification, protein turnover, chaperones” (942, 10.14%), and “Carbon hydrate transport and metabolism” (913, 9.83%).

### Kyoto Encyclopedia of Genes and Genomes-Based Pathway Assignment of Unique Sequences

**FIGURE 4:**
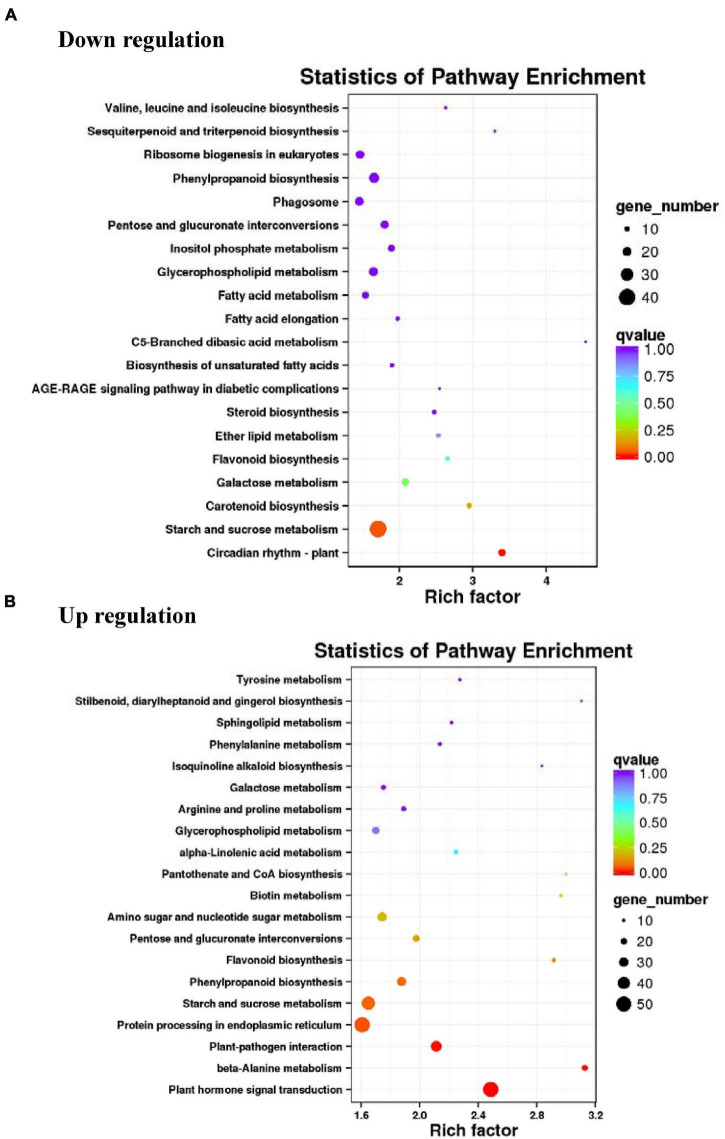
Kyoto Encyclopedia of Genes and Genomes (KEGG) pathway analysis of differentially expressed genes (DEGs) in RNA-seq. Top 20 pathways were characterized among the upregulated **(A)** and downregulated **(B)** DEGs.

Overall, 12,416 unique sequences could be annotated with KEGG terms, and out of these 1,192 were assigned to the biosynthesis of the secondary metabolite pathway. Expectedly, metabolic pathways were well represented among *L. sprengeri* unique sequences (Figure 4 and Supplementary Table 4). Most of the downregulated genes in white vs. pink petals were mapped to the pathways of circadian rhythm-plant, starch and sucrose metabolism, carotenoid biosynthesis, galactose metabolism, and flavonoid biosynthesis (Figure 4A) while most of the upregulated genes were mapped to plant hormone signal transduction, beta-alanine metabolism, phenylpropanoid biosynthesis, starch and sucrose metabolism, plant-pathogen interaction, and flavonoid biosynthesis (Figure 4B). Transcripts encoding flavonoid biosynthesis enzymes detected in our Illumina sequencing data set are tabulated under Supplementary Table 5.

### Anthocyanin Biosynthesis Genes and Pigments Are Repressed in White vs. Pink Petals

**FIGURE 5:**
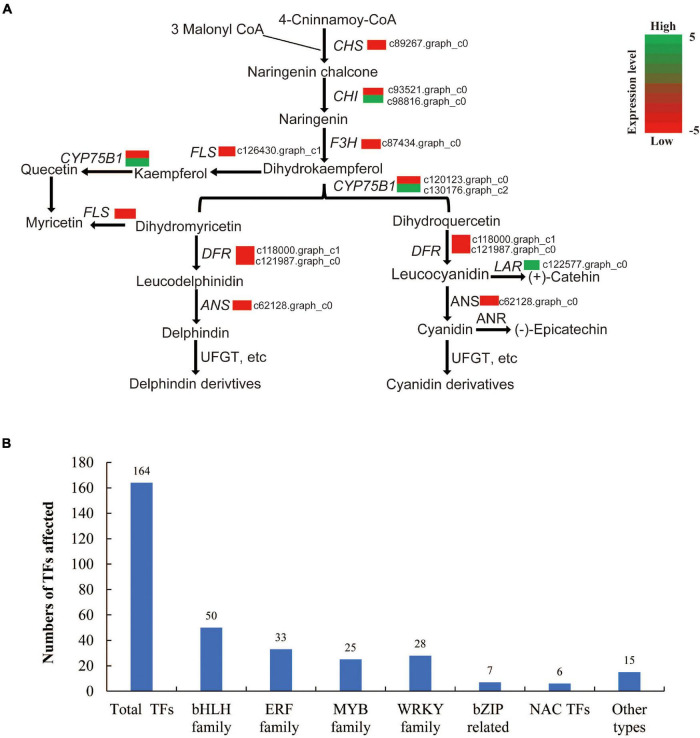
Genes involved in flavonoid biosynthesis and total transcription factors (TFs) identified in RNA-seq. **(A)** Genes involved in flavonoid biosynthesis. **(B)** An overview of the total number TFs in this study.

It is hypothesized that flower pigmentation is mainly related to the genes involved in flavone, anthocyanin, and flavonoid biosynthesis pathways. After searching against the combined functional annotation, a total of 41 key structural genes (encoding pigment biosynthesis enzymes) were identified and 8 of them showed significant downregulation in white vs. pink petals. We compared the pigment metabolic process and its main branches (with core metabolites and enzymes) to figure out why some steps in the anthocyanin biosynthesis pathway are blocked in the white petals (Figure 5A). This included *CHS*, *F3H*, *FLS*, *ANS*, and *DFR*. The expression of leucoanthocyanidin reductase (*LAR*) was significantly upregulated in white vs. pink petals. However, among the unigenes matched to chalcone isomerase (*CHI*), c93521.graph_c0 was downregulated and c98816.graph_c0 was upregulated. Similarly, among the unigenes for caffeoyl-flavonoid 3′-monooxygenase (CYP75B1), c120123.graph_c0 was downregulated and c130176.graph_c2 was upregulated (Figure 5A). Interestingly, increased Ca in white vs. pink petals can be attributed to a higher LAR enzyme level. Based on the analysis of DEGs involved in anthocyanin biosynthesis, we may figure out that the DEGs of anthocyanin biosynthesis may give further evidence to interpret mechanisms, which may cause the different accumulations of anthocyanin-related metabolites and finally led to the different color forms in *L. sprengeri*, all the other core metabolites were also detected in the pink petal extracts (Figure 2B).

### Annotation of Transcription Factor Gene Families

Plant TF database was searched for annotating the detected *L. sprengeri* transcripts as putative transcriptional regulators (Pérez-Rodríguez et al., 2010). In our study, a total of 164 transcripts were predicted as TFs, which mainly belonged to 6 families, including 50 bHLH, 33 ethylene responsive factor family (ERF), 25 MYB, 28 WRKY, 7 bZIP, and 6 NAC family TFs (Figure 5B). Of these, we identified three R2R3 TFs (c128273.graph_c0, c126227.graph_c2, and c105793.graph_c0), which were greatly repressed in white vs. pink petals. On the other hand, the expression of eight MYB family TFs (c120875.graph_c0, c124022.graph_c2, c118923.graph_c0, c116915.graph_c0, c123390.graph_c0, c119914.graph_c0, c107135.graph_c0, and c102137.graph_c0) showed 4-fold or greater changes in white vs. pink petals while others are MYB-related proteins with unknown annotations, suggesting a potential involvement of these eight MYB TFs in the gene regulation of the expression of biosynthesis genes for pigment enzymes controlling contrasting factors, such as anthocyanin biosynthesis, petal color development, photosynthesis, and multiple biological processes in plants that may compensate for the petal color variations as well.

To analyze the relationships between those three R2R3 MYB TFs and the other eight MYB TFs, a phylogenetic analysis of the three R2R3 MYB TFs and the other eight MYB TFs identified was employed in our study. The results suggest that the three R2R3 are phylogenetically related while others belong to other types (Supplementary Figure 3). A qPCR analysis of these transcriptional factors demonstrated that all the 3 R2R3 are downregulated and the expression of the other 11 TFs is upregulated, and the obtained fold changes were consistent with those in the RNA-seq assay (Supplementary Figure 4).

### Genes Involved in Steroid Biosynthesis and Brassinolide Levels Are Repressed While pH Is Higher in White Petals

**FIGURE 6:**
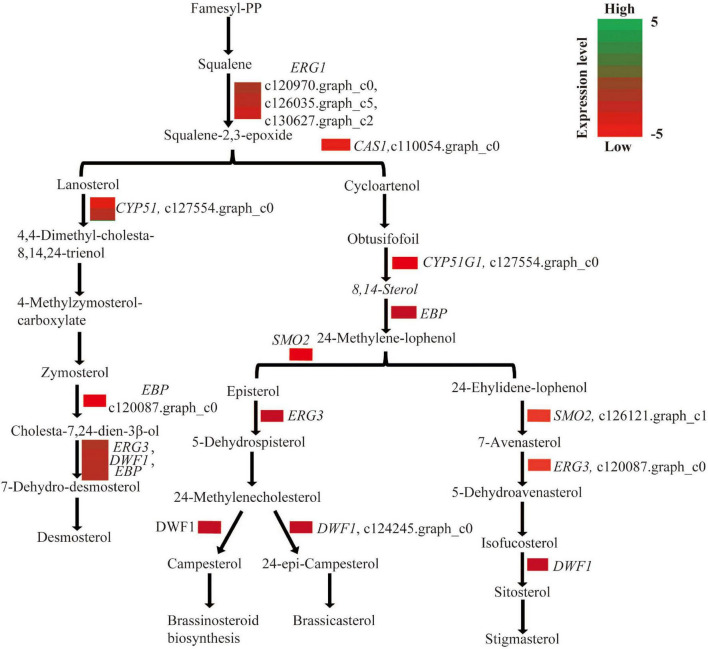
Genes involved in brassinosteroid biosynthesis were repressed in white vs. pink petals.

In this study, a total of 10 genes, including *ERG1* (squalene monooxygenase), *CAS1* (cycloartenol synthase), *CYP51* (sterol 14-alfa-demethylase), *EBP* (cholestenol delta-isomerase), *SMO2* (alpha-monomethylsterol monooxygenase), *ERG3* (delta-7-sterol 5-desaturase), and *DWF1* (sterol reductase), which are all involved in steroid biosynthesis, were repressed in white vs. pink petals (Figure 6). To investigate the brassinosteroid contents in white and pink petals, HPLC-MS/MS was employed to compare the BL content between them. The BL content in white petals was about nine times lower than that in pink petals of *L. sprengeri* (Supplementary Figure 2). The pH inside white petals is significantly higher than that inside pink petals (Supplementary Table 13).

### Gene Expression of Flavonoid Biosynthesis Genes

As the flower color in plants is mainly determined by the anthocyanin pathway, the expression levels of a few genes identified in our RNA-seq were quantified with a qPCR assay. Eight genes, *LsCHS*, *LsCHI*, *LsF3H*, *LsCYP75B1*, *LsFLS*, *LsDFR*, *LsANS*, and *LsLAR*, from the flavonoid biosynthetic pathway, were selected for qPCR validation. Most of the genes were greatly suppressed in white petals, and only the expression level of *LsLAR* was upregulated in white vs. pink petals (Supplementary Figure 5 and Supplementary Table 10). The expression levels of *LsDFR*, *LsCHS*, *LsANS*, and *LsFLS* are supposed to be directly related to flower color development (Shan et al., 2020).

## Discussion

*Lycoris sprengeri* is an important herb, and the *Lycoris* genus has about 20 species, of which ten are commonly grown in China and Southern Asia (Shi et al., 2019). Previous transcriptomic studies relevant to the Amaryllidaceae family investigated *L. aurea* (Wang et al., 2013), *L. longituba* (Wang et al., 2013; Li et al., 2020), *Allium cepa* (Kim et al., 2015), *Narcissus pseudonarcissus* (Singh and Desgagné-Penix, 2017), and *L. sprengeri* (Chang et al., 2011). Hereby, we profiled the transcriptome and metabolite profiles of white and pink petals of *L. sprengeri*. The transcriptome analysis was used to identify the differentially regulated genes that could explain the color differences and hence control flower coloration (Supplementary Tables 3, 4). In comparison with the transcriptomic analysis in other *Lycoris* species, this study has better gene coverage and sequence assembly performance in total sequences and total unigenes (Wang et al., 2013; Park et al., 2019; Supplementary Tables 11, 12).

Most unigenes were associated with molecular function terms, such as “binding” and “catalytic activity,” cellular component terms such as “cell” and “cell parts” while the biological process terms mainly are “cellular process” and “metabolic processes” (Figure 3B). These results are consistent with a previous transcriptome study on *L. aurea* (Wang et al., 2013). Most unigenes in the study were reported to be involved in “translation, ribosomal structure, and biogenesis,” “post-translational modification,” “protein turnover, chaperones,” and “general functional prediction only,” “binding and catalytic activity,” “cell,” “cell parts,” and “organelle and cellular and metabolic processes” (Wang et al., 2013). However, we obtained more unigenes in our transcriptomic study and present a more detailed GO annotation. Chang et al. (2011) also reported similar results for the GO annotation for the transcriptome of *L. sprengeri*. COG/eggNOG annotation analyses revealed that the pathways that were annotated to the identified unique transcripts belonged to lipid metabolism, transcriptions, signal transduction, translational, protein turn over and chaperone, and ribosomal biogenesis (Figures 3C,D). This suggested that the major category in COG annotation was “General function” and “lipid metabolism” while in eggNOG the largest metabolic pathway was “post-translational modification, protein turnover and chaperones.” These categories may be quite important for the color development in *L. sprengeri* petals. The KEGG analysis revealed the enrichment of color development-related flavonoid biosynthesis pathway in both upregulated and downregulated genes (Figure 4 and Supplementary Table 9). Furthermore, 20 color-related DEGs were identified between white and pink petals and associated with pigments: anthocyanins, flavonoids, and flavonols, which were considered to contribute to the flower color and their levels could determine the final flower color variation from pink to white (Tanaka et al., 1998; Aida et al., 2000). In the following section, we are going to discuss the candidate genes, which may be responsible for the petal coloration.

### Candidate Genes Responsible for Loss of Pink Color in White Petals of *Lycoris sprengeri*

Anthocyanins are a type of flavonoids implied in the formation of different non-white petal colors (Tanaka et al., 1998; Zhao and Tao, 2015). Common enzymatic reactions in the anthocyanin pathway are catalyzed by *CHS*, *F3H*, and *CHI* while other genes are responsible for specific metabolite synthesis in the anthocyanin pathway (Xue et al., 2021). It is well known that Del and Cy are reported to be the direct reason for a color difference in petals (Lou et al., 2014). Our transcriptome analysis demonstrated that anthocyanin biosynthetic activities were repressed in white petals due to the repressed transcript levels of *CHS*, *FLS*, *F3H*, *ANS*, and *DFR* (Figure 5A). Gene expression repression of flavonoid biosynthesis enzyme genes was validated in white petals through qPCR to validate the accuracy and reproducibility (Supplementary Figure 5). These genes are reported to be involved in various biosynthesis stages of the anthocyanin biosynthesis process. For example, *ANS* is an α-ketoglutarate-dependent dioxygenase acting downstream of the anthocyanin biosynthesis pathway, which mediates the catalysis of anthocyanin formation from α-ketoglutaric acid and Fe^2+^ (Tan et al., 2013). *CHS* (encodes a chalcone synthase) and *F3*’*H* (encodes a flavanone 3-hydroxylase) are also important genes involved in the regulation of flower color and catalyzed the early enzymatic reactions in the flavonoid biosynthesis pathway (Lou et al., 2014). *DFR* (encodes dihydroflavonol reductase) is an enzyme, which reduces dihydroflavonols to colorless leucoanthocyanidins. This in turn is converted into colored anthocyanidins by *ANS*. These genes and their corresponding catalytic reactions might be occurred by key genes and speed-limiting steps leading to the loss of anthocyanins in white petals. Although the *DFR* gene effectively restricts the Cy metabolism branch, Ca was found in white petals, suggesting that white petals were without the lack of anthocyanin metabolism downstream genes. Meanwhile, the content of Km in white petals was higher than that in pink petals, indicating that the anthocyanin biosynthetic pathways (ABP) in white petals is restrained and some amounts of Km are accumulated (Figure 2B). The ANS gene also plays a key role in the ABP pathway, which could catalyze the synthesis of colorless anthocyanins into colored anthocyanins (Xue et al., 2019). The reduced expression of ANS gene might be a factor in its inability to accumulate anthocyanins in white petals (Figure 5A).

### Metabolic Compounds Associated With Flower Coloration in *Lycoris sprengeri*

The metabolic regulation related to the formation of flower color is a complex metabolic pathway, which is regulated by the key genes of several branch metabolic pathways and regulatory factors. It was difficult to obtain an absolute coloration between the genes and the corresponding metabolites. The anthocyanin composition and content and copigment flavonol content in petals were analyzed to understand the biochemical basis of different flower colors and to predict the genetic differences. In this study, the metabolic profiling of anthocyanin-related metabolites was performed, which showed that Ep, Cy, and Del were significantly lower while Ca, Km, and Er were significantly more in white petals than in pink petals (Figure 2B). Lower Ep levels in white petals suggested that the color pigment Cy may either be present at a significantly lower level or maybe stable for a short time in white petals compared to pink petals. In the latter case, it is probable that the unstable Cy is converted to colorless Ep, which would prevent the production of a color pigment from Cy *via* later glycosylation and other reactions. Interestingly, reduced products downstream of dihydromyricetin (DFR substrate) in the Del synthesis pathway (a substrate for the DFR enzyme) were detected in white vs. pink petals (Figure 5A), suggesting that *DFR* was the most likely target for Del suppression in the white petals of *L. sprengeri* (Figure 2B). *FLS* (encodes flavonol synthase) acts as a coregulator for controlling the amounts of dihydroflavonols for synthesizing the intermediates in the production of both the colored anthocyanins together with *DFR*. It was suggested in a recent study that the contents of Cy can be correlated to the expression levels of *FLS* and *DFR* (Lou et al., 2014). Thus, the repressed levels of *FLS* together with *DFR* may produce lower anthocyanin (Davies et al., 2003). A similar mechanism may act in white petals due to the repression of these enzyme genes.

### Mechanism of Flower Coloration in *Lycoris sprengeri*

Even though the contents of some anthocyanins are relatively high in white flowers (Figure 2B), the type of flower color could be affected by other conditions including pH values such as the color form of anthocyanins is red in acidic pH, colorless or white in neutral or nearly neutral pH, and blue in alkaline pH. The pH values of pink petals are significantly lower than those in white petals (Supplementary Table 13). Four anthocyanin tautomers in different pH values: alkali blue quinone A, red–yellow molten cation AH+, colorless false base B, and colorless chalcone C could cause different colorful flower forms, and the pH can influence the three balance conversions between them (Zhao and Tao, 2015). The colorless false base B and colorless C may be the major form in white petals, and red-yellow molten cation AH+ represents the major anthocyanin in pink petals (Zhao and Tao, 2015).

The expression of structural genes in anthocyanin biosynthesis and brassinosteroid biosynthesis-related genes may be controlled by transcriptional regulators (Wang et al., 2021). Previously, 51 TFs were recognized as flower specific and belonged to MADS, MYB-related, NAC, and others (He et al., 2010). However, there is still no standardized reference genome for *Lycoris* and therefore TF annotation information is limited. We cataloged TFs in our petal transcriptome based on publicly available data sets (Figure 5B). Three major groups of TFs, belonging to bHLH, R2R3 MYB, and WD40 families, were predominantly reported to regulate genes in the anthocyanin biosynthesis pathway across the majority of plant species (Stracke et al., 2001; Allan et al., 2008). MYB proteins have been implicated in secondary metabolism, development, signal transduction, and disease resistance in plants (Jin and Martin, 1999). MYB TF contains 339 TFs reported in Arabidopsis (Feller et al., 2011), and is associated with the anthocyanin biosynthesis pathway. In this study, three R2R3 MYB TFs were greatly repressed in white vs. pink petals. R2R3 MYB are among the MYB TF types considered to be closely related to anthocyanin metabolism and regulation (Zhao and Tao, 2015). These three phylogenetically related R2R3 MYB TFs were grouped distinctly from the other eight MYB TFs (Supplementary Figure 3). Their relative expression levels were further confirmed to be consistent with their expression pattern in RNA-seq (Supplementary Figure 4). The evolutionary transition to white petals *via* multiple losses of floral anthocyanin production is associated with the mutation of an R2R3 MYB transcriptional activator (Gates et al., 2018). It is thus highly likely that the repression of R2R3 MYB TFs in our study may lead to white color petals in comparison with pink petals.

In addition, the genes involved in steroid and BR biosynthesis pathways were all downregulated in white petals (Figure 6). BR treatment enhances the coloration and increases the anthocyanin content in grape berries from veraison onset to full veraison (Zheng et al., 2020). Therefore, the reduced transcript levels of genes involved in the steroid biosynthesis may lead to lower BR contents in white petals of *L*. *sprengeri*, which may, in turn, affect the anthocyanidin biosynthesis and result in the fainter color variations of *Lycoris* petals. The most potent BR, BL, is produced by the campesterol (Wei and Li, 2020) in the BR biosynthesis pathway that could further affect the phytohormone, flowering time, plant growth, and seed yield in plants. Consistently, the BL content in white petals was about nine times lower than pink petals (Supplementary Figure 2). We, therefore, confirm that the suppressed levels of genes involved in steroid biosynthesis could lead to lower levels of BL contents and subsequently lead to reduced levels of anthocyanin biosynthesis in white petals. It was also reported that BR could increase the anthocyanin biosynthetic activity by an upregulation of the late anthocyanin biosynthetic genes, such as *DFR*, *leucoanthocyanidin dioxygenase* (*LDOX*), and *UDP-glucose: flavonoid-3-O-glucosyl transferase* (*UF3GT*) in Arabidopsis while the anthocyanin accumulation was reduced in a BR-gene deficient mutant that may be caused by the repressed expressions of late anthocyanin biosynthetic genes (Yuan et al., 2015). In our study, the anthocyanin accumulation levels are decreased in white petals, and as a result, the expression of BR biosynthesis pathway-related genes is greatly suppressed as well as the structural genes and downstream genes suggest that the accumulation of anthocyanins may be one of the reasons for regulating the biosynthetic activities in the BR biosynthesis pathway. However, a variation in the different color forms of petals is regulated by multiple factors, and the BR pathway may be one of the factors affecting anthocyanin accumulation and finally lead to different color types. However, we may need more experimental data to support the relationship between the BR biosynthesis and flower color development in plants.

The mechanism of different petal colorations in *L. sprengeri* was speculated. Overall, pink petals contained more types and higher levels of intermediate compounds in the flavonoid biosynthesis pathway compared to white petals, with a few exceptions. We assume that because of ABP’s restrained effects in white petals, the upstream flux must flow into other branches of the flavonoid metabolic route. The underlying mechanism is probably more complex is what is described here. The mechanism would be clarified by identifying a characteristic structure of genes and by examining how the occurrence of TFs, transporters, or miRNA affected the flowers in our future work. Exploring the molecular mechanism of petal coloration might help apply genetic engineering to produce other novel colors of *Lycoris* spp.

## Conclusion

Taken together, we can hypothesize that the regulation of petal color may be determined by the expression levels of steroid and anthocyanin biosynthesis genes regulated by MYB TFs. Further studies are needed to determine whether the three repressed R2R3 MYB TF genes (in white petals) could be related to the regulation of repressed BR and anthocyanin genes in white petals. Specifically, quantifying the level of these genes and pigments in the mutants (white petals) of these TFs and its further functional characterization in *L. sprengeri* or of orthologous genes in model species can be undertaken using the CRISPR genome editing technique. Altogether, our transcriptome and metabolite quantification approach provides evidence for the genes putatively responsible for the lack of color phenotype in the white petals. This could provide valuable information for breeders to produce the different colors of *L. sprengeri* petals in horticultural research.

## Data Availability Statement

RNA-Seq data obtained hereby have been uploaded to the Sequence Read Archive (https://www.ncbi.nlm.nih.gov/sra/SUB9202653/overview) and can be accessed with accession number PRJNA714286.

## Author Contributions

Q-ZL and JZ designed the experiments. FY, Q-ZL, and JZ got the funding. FY, DD, and C-HL performed the experiments. Q-ZL, DD, C-HL, Y-HZ, M-XC, TS, L-XW, and JZ analyzed the data. FY, Q-ZL, C-HL, DD, and JZ wrote the manuscript with contributions from all other authors.

## Conflict of Interest

The authors declare that the research was conducted in the absence of any commercial or financial relationships that could be construed as a potential conflict of interest.

## Publisher’s Note

All claims expressed in this article are solely those of the authors and do not necessarily represent those of their affiliated organizations, or those of the publisher, the editors and the reviewers. Any product that may be evaluated in this article, or claim that may be made by its manufacturer, is not guaranteed or endorsed by the publisher.

## Footnotes

1
http://trinityrnaseq.sourceforge.net/

2
ftp://ftp.ncbi.nih.gov/blast/

3
http://www.uniprot.org/

4
http://www.genome.jp/kegg/

5
http://www.ncbi.nlm.nih.gov/COG/

6 http://www.ncbi.nlm.nih.gov/COG/; https://ftp.ncbi.nih.gov/pub/COG/

7
http://eggnogdb.embl.de/

8
http://pfam.xfam.org/

9
http://www.geneontology.org/

10
http://soap.genomics.org.c/soapaligner.html

11
https://submit.ncbi.nlm.nih.gov/subs/sra/SUB9202653/overview

## Supplementary Material

Supplementary Figure 1 Principal component analysis (PCA) comparison between three pink and three white petals.

Supplementary Figure 2 Comparison of brassinolide (BL) contents in white and pink petals in *Lycoris sprengeri.*

Supplementary Figure 3 Phylogenetic tree analysis of MYB transcription factors (TFs) in *L. sprengeri*.

Supplementary Figure 4 Quantitative PCR (qPCR) analysis of MYB TFs in white and pink petals in *L. sprengeri*.

Supplementary Figure 5 qPCR analysis of genes involved in flavonoid biosynthesis identified in RNA sequencing (RNA-seq). *LsUbiquitin* (c105178.graph_c0) was used as an internal control. The expression levels of genes in WT plants were set to one.

Supplementary Table 1 Quality control of RNA sequencing (RNA-seq).

Supplementary Table 2 Overview of transcriptome data assembly.

Supplementary Table 3 Data assembly results.

Supplementary Table 4 Kyoto Encyclopedia of Genes and Genomes (KEGG) pathway analysis.

Supplementary Table 5 Total differentially expressed genes (DEGs) identified in this study.

Supplementary Table 6 Metabolic profiles of flavonoid biosynthesis-related metabolites in this study.

Supplementary Table 7 Summary of numbers of single nucleotide polymorphism (SNP) in samples analyzed in pink and white petals.

Supplementary Table 8 Total SNPs in this study.

Supplementary Table 9 Simple sequence repeat (SSR) analysis in this study.

Supplementary Table 10 Primers used for quantitative PCR (qPCR) analysis in this study.

Supplementary Table 11 Comparison of the transcriptome analysis in recent studies.

Supplementary Table 12 All annotated unigene numbers from different databases.

Supplementary Table 13 pH measurements in white and pink petals.
