# Supplementary figures and images for: Comprehensive Transcriptome and Metabolic Profiling of Petal Color Development in Lycoris sprengeri
Source: Front Plant Sci. 2021 Dec 3;12:747131. doi: 10.3389/fpls.2021.747131 (PMC8678534; doi:10.3389/fpls.2021.747131)

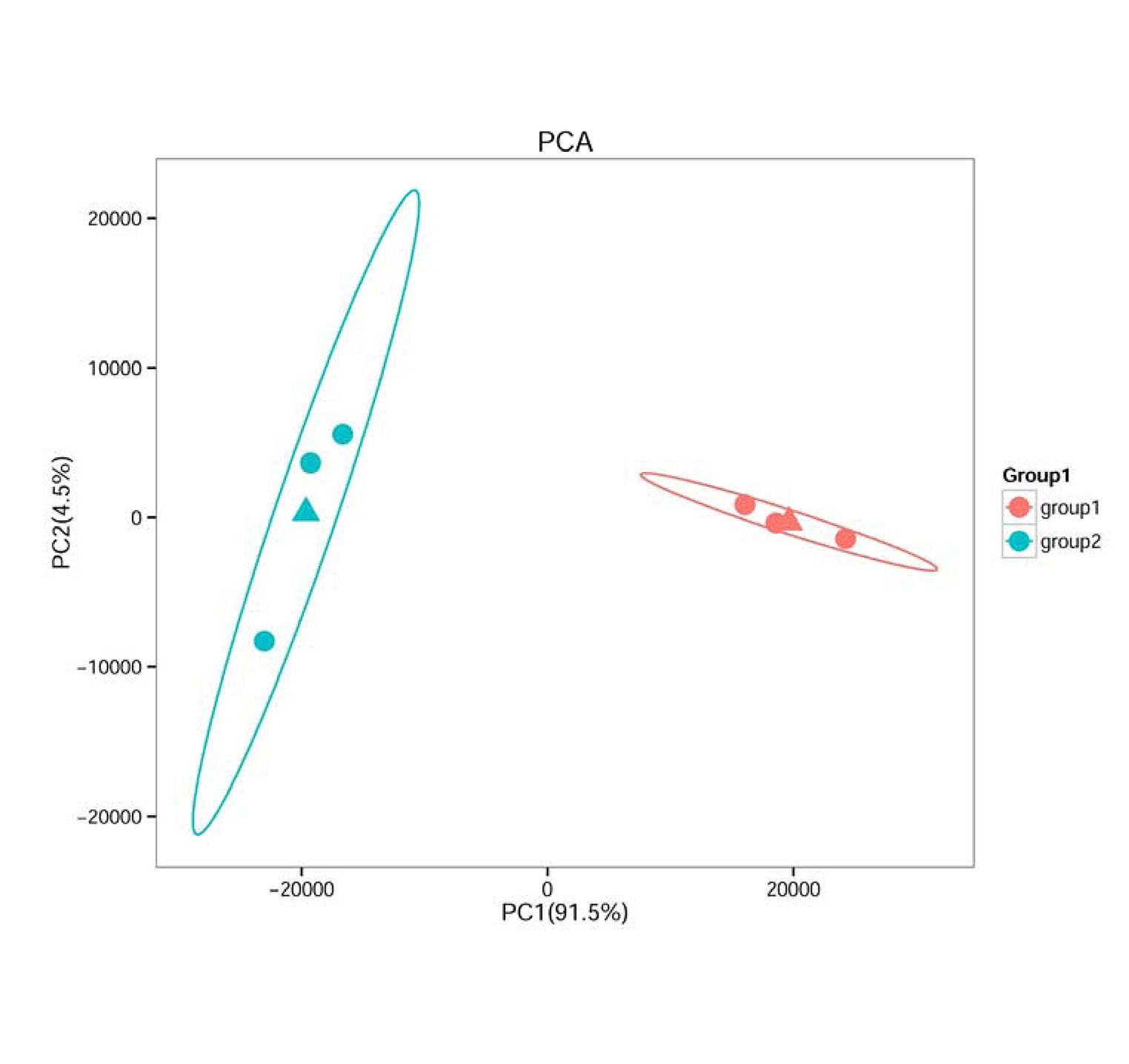

Supplement: Supplementary Figure 1 — Principal component analysis (PCA) comparison between three pink and three white petals. [file Image_1.JPEG]

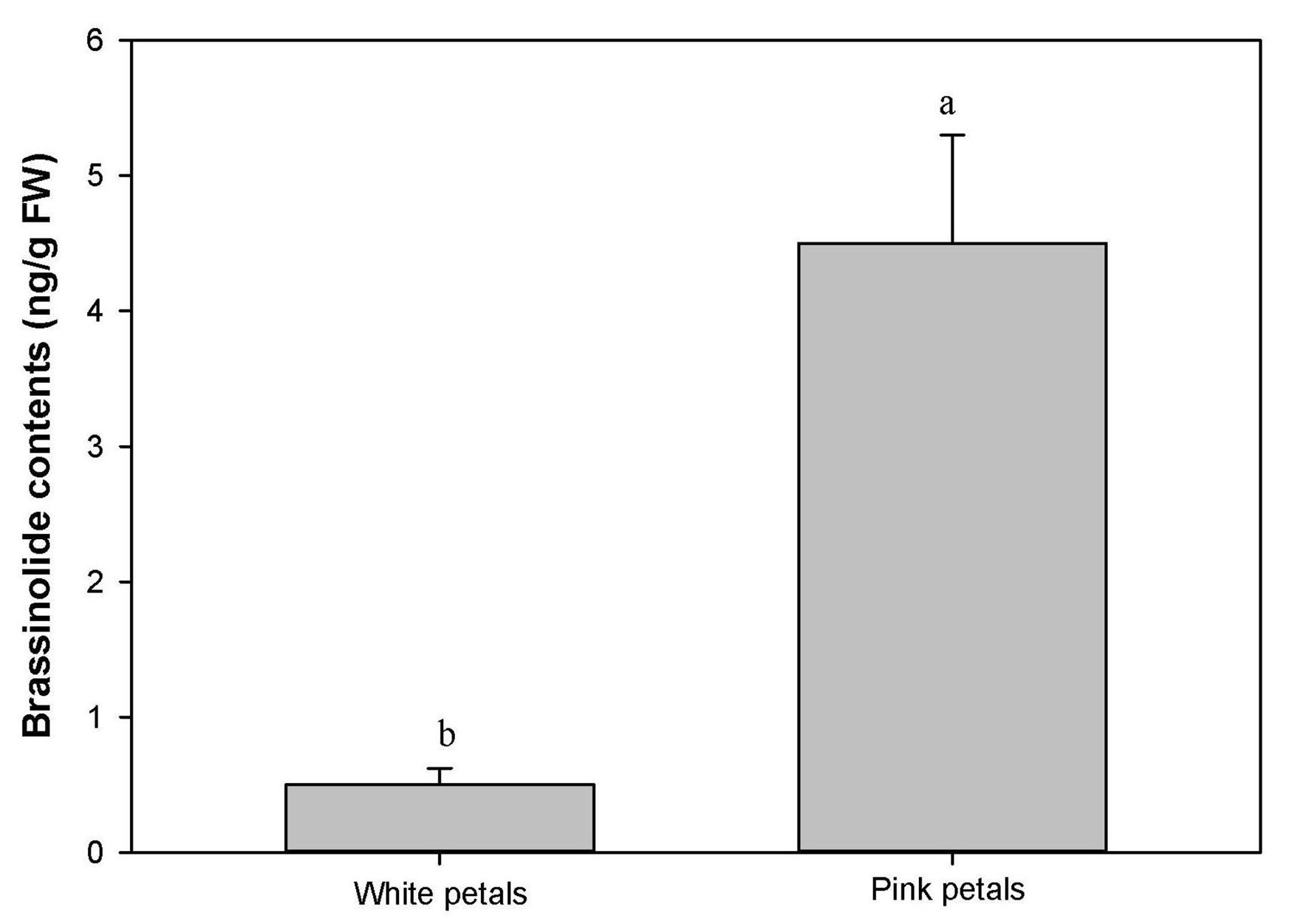

Supplement: Supplementary Figure 2 — Comparison of brassinolide (BL) contents in white and pink petals in Lycoris sprengeri. [file Image_2.JPEG]

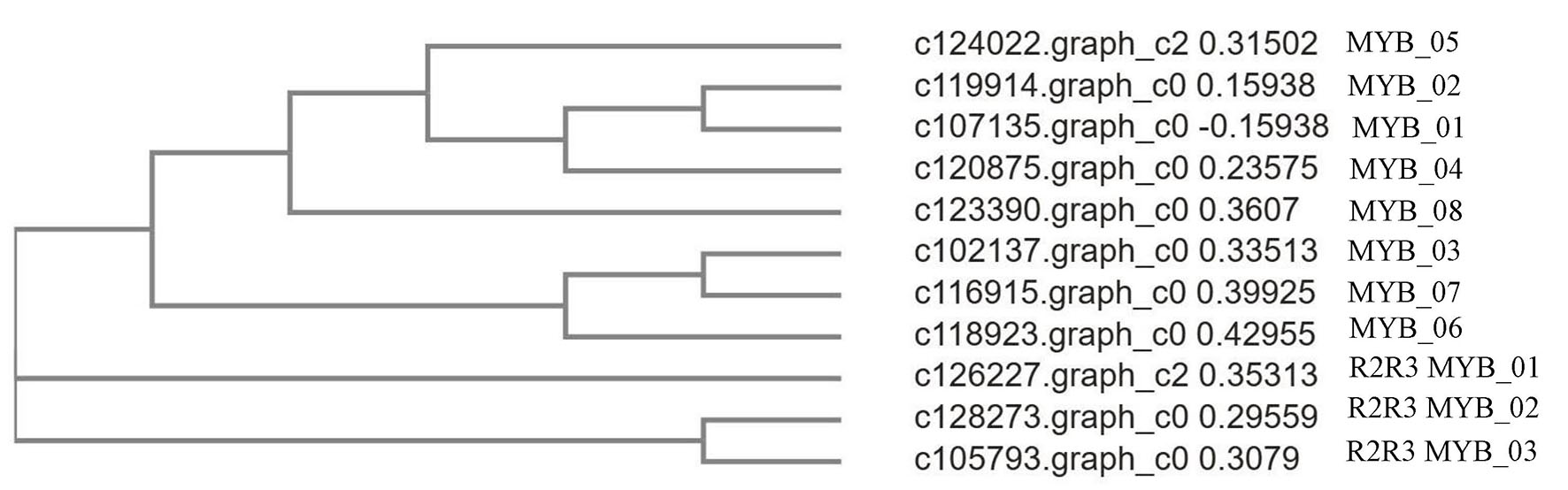

Supplement: Supplementary Figure 3 — Phylogenetic tree analysis of MYB transcription factors (TFs) in L. sprengeri. [file Image_3.JPEG]

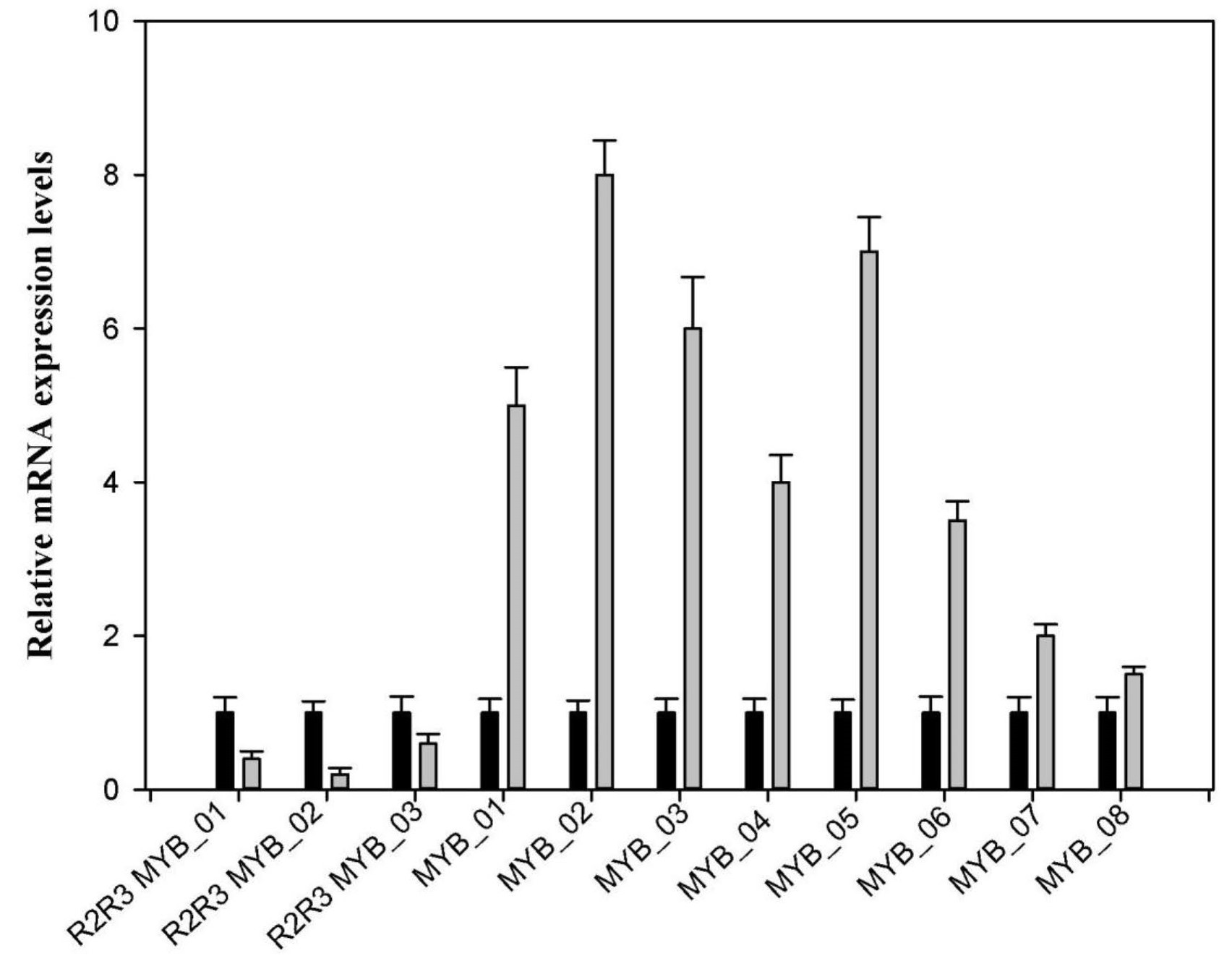

Supplement: Supplementary Figure 4 — Quantitative PCR (qPCR) analysis of MYB TFs in white and pink petals in L. sprengeri. [file Image_4.JPEG]

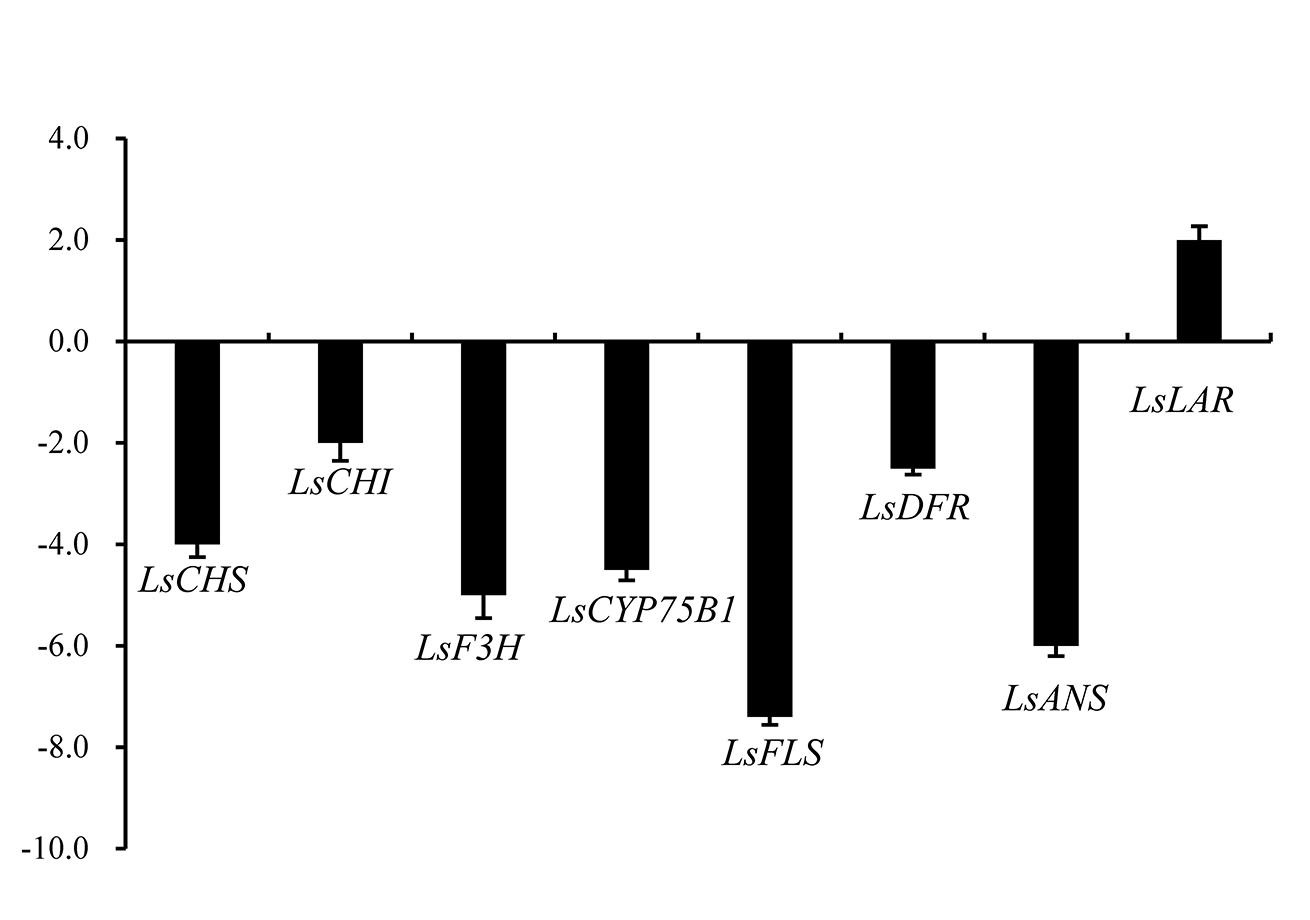

Supplement: Supplementary Figure 5 — qPCR analysis of genes involved in flavonoid biosynthesis identified in RNA sequencing (RNA-seq). LsUbiquitin (c105178.graph_c0) was used as an internal control. The expression levels of genes in WT plants were set to one. [file Image_5.JPEG]
